# Wetland Soil Phosphorus and Phosphorus Sorption Capacity in Wastewater-impacted and Unimpacted Wetlands, NH USA

**DOI:** 10.21203/rs.3.rs-10539732/v1

**Published:** 2026-09-10

**Authors:** Shannon Stroble, Catherine Owen Koning

**Affiliations:** Franklin Pierce University; Franklin Pierce University

**Keywords:** Wetlands, Phosphorus Saturation Ratio (PSR), Soil Phosphorus Storage Capacity (SPSC), Total Phosphorus (TP), Treated Wastewater, Phragmites australis

## Abstract

The long-term impact of treated wastewater effluent on the soil in a 7.3 ha emergent marsh in southwestern New Hampshire, USA, known locally as Beaver Dam Marsh (BDM), was evaluated by comparison to four nearby unaffected wetlands. We hypothesized that 40 years of wastewater input would result in a higher phosphorus saturation ratio (PSR), lower soil phosphorus storage capacity (SPSC), and higher total phosphorus (TP) in the soils of BDM. Samples of the surface soil and the subsoil were collected and analyzed for TP via colorimetric analysis and extractable phosphorus, aluminum and iron via Mehlich-3 extraction paired with Inductively Coupled Plasma (ICP) spectroscopy.

The surface soil of BDM showed no significant difference in PSR (p = 0.90) and SPSC (p = 0.79) while the subsoil exhibited a significantly lower PSR (p = 0.029) than the unaffected wetlands. Additionally, there was a significantly lower TP by dry mass in BDM surface soil than the reference wetlands (p = 2.3 × 10^−4^). The volumetric TP was also lower in BDM; however, this difference was not statistically significant. Phosphorus sequestration within the organic biomass of the invasive *Phragmites australis* is likely a contributing factor to the lower dry-mass TP. On-going inputs of treated wastewater, low soil pH, as well as saturated conditions, appear to be interfering with formation of inorganic phosphorus complexes with iron and aluminum. Despite some spatial variation within BDM, the wetland soil appears unable to retain any additional phosphorus from the ongoing inputs of treated wastewater.

## INTRODUCTION

Wetland ecosystems are well known for their ability to filter and transform pollutants. Many studies have documented this function, particularly for heavy metals (e.g., Rai 2008), sediments (Knox et al. 2008), nitrogen (Fisher and Acreman 2004) and other pollutants. The impact of wetland ecosystems on phosphorus inputs, however, has been variable (Kadlec 2019; Skinner 2022). Phosphorus inputs to surface water systems contribute to eutrophication. Globally, 57% of lakes are considered eutrophic (Wang et al. 2025), 43% in the United States (USEPA 2022), and 37% in New Hampshire, USA (NHDES 2022b).

Researchers have examined the efficacy of constructed wetlands for phosphorus removal (e.g., Wood et al. 2008), and compared them to natural wetlands (e.g., Hogan et al. 2004; Duff et al. 2009). Rates of phosphorus removal depend on soil and vegetation type and density, hydraulic retention times, and water level fluctuations (Kadlec 2019; Baustian et al. 2018; VanZomeren et al. 2020).

Wetlands remove available phosphorus from the water column through several different pathways; refer to Kadlec (2019) and Reddy et al. (2005) for excellent summaries. Chemical pathways of phosphorus removal include the reaction of soluble phosphate with cations such as aluminum and iron in acid soils, and calcium and magnesium in alkaline soils. Under oxic conditions, phosphate species can adsorb onto or within cation complexes, which can form a solid precipitate, such as apatite and hydroxylapatite (Reddy et al. 2005). When conditions become anoxic, the cation complexes will dissociate, releasing phosphorus back into the water column. Anoxic conditions are most common in wetland soils, especially when soils are waterlogged and there is little flow (Liu et al. 2017). Because water levels and flow rates vary through the season and in different parts of the same wetland, this mechanism of phosphorus removal is inconsistent across time and space and is often temporary (Reddy et al. 2005). The ability of the soil to adsorb additional phosphorus can be measured by phosphorus saturation ratio (PSR), which is the ratio of total extractable phosphorus to the total extractable cations (aluminum and iron in acidic soils, and magnesium and calcium in alkaline soils). This can also be expressed as soil phosphorus storage capacity (SPSC), which estimates the additional amount of phosphorus a soil can retain before reaching an environmental risk threshold.

Biological processes are responsible for the long-term storages of phosphorus in wetlands, which has been shown to comprise > 90% of the phosphorus pool in enriched wetlands (Kadlec 2019; Reddy et al. 2005). Organic phosphorus taken up by plants is then incorporated into the soil at the end of the growing season. Soil accretion especially in histosols or mineral histic soil horizons can be a significant storage of phosphorus (Kadlec 2019). Sedimentation of particulate phosphorus is possible particularly in densely vegetated wetlands (Brown and Stark 1989; Baustian et al. 2018).

Wetlands that have been subjected to continuous influxes of anthropogenic phosphorus have been shown to provide long-term storage of phosphorus (Dunne et al. 2007). The surface soil (0–10 cm) stored 87% of the phosphorus in a Florida wetland subject to agricultural inputs (Dunne et al 2007). In some cases, however, the ability of the wetland soils to retain phosphorus decreases over time, and the wetland can become a source of phosphorus (Jeng and Hong 2005).

Wetlands subject to treated wastewater effluent essentially act as tertiary treatment or “polishing” stages. The focus of this study is a 7.3 ha emergent marsh, known locally as Beaver Dam Marsh (BDM) which is part of a larger forested wetland complex, downstream from the Franklin Pierce University wastewater treatment facility (WWTF) (Fig. 1). For over 40 years, the WWTF discharged treated effluent into a headwater stream, which flowed into the wetland complex; this stream eventually flows into a 78-ha shallow lake, which was declared impaired in 2010 and is considered eutrophic (Comprehensive Environmental Inc. 2014). As a result, the New Hampshire Department of Environmental Services (NHDES) required the University to install a groundwater discharge system in 2009, in addition to other upgrades. The effluent is now piped underground from the WWTF into a Rapid Infiltration Bed (RIB) sand filter, into the groundwater, which then discharges south to the lake and north into BDM. This groundwater discharge system has had hydrologic, chemical and biodiversity impacts on adjacent forested wetlands (Koning and Bell, 2014) and on BDM, as demonstrated by higher specific conductance and higher chloride levels (Brandt 2022; Koning and Stroble 2022); chloride is added to the drinking water of the University as part of an effort to remove excess iron. Groundwater in New Hampshire, particularly in this area, is known to be high in iron as result of underlying bedrock (Moore 2004), which in this area is metasedimentary Upper Rangely formation schist (Lyons et al. 1997).

Modeling of the hydrologic and phosphorus inputs (Balke et al. 2018) suggested BDM was still releasing phosphorus, even several years after the cessation of direct discharge. This led to the goal of this study, which is to determine whether the soil in BDM is saturated with phosphorus, and to compare the PSR and the SPSC in BDM with similar, unimpacted wetlands nearby. Sites for available phosphorus sorption are expected to be already taken up in this wastewater impacted wetland, and years of high phosphorus loading are likely to have resulted in higher total phosphorus in the organic layers. We hypothesized that, when compared to the unimpacted wetlands (1) BDM would have higher PSR and lower SPSC, and (2) BDM would have significantly higher total phosphorus in the organic layers of the surface soils.

## METHODS

### Site Description

The five wetlands chosen for this study were all located in the upper watershed of the Miller’s River, a tributary of the Connecticut River, in Jaffrey and Rindge, New Hampshire, USA (Fig. 2). All five wetlands are small (less than 12 ha) and are associated with second- or third-order streams. All are open, unforested wetlands dominated by grasses, sedges, and low shrubs (Table 1), classified by the National Wetland Inventory as palustrine, with emergent, persistent vegetation or woody shrubs, seasonally flooded or saturated, some sections being semi-permanently flooded. They are classified as borohemist ponded soils. All five sites show hydrologic impacts from beaver dams or roads. The catchment areas for each wetland are all forested, with less than 10 buildings, with the exception of BDM, which has a small university (< 1000 students) in its watershed.

As described earlier, BDM continues to receive treated wastewater via groundwater flow from the RIB (Fig. 1). Based on monitoring wells around the RIBs, the existing treated effluent continues to demonstrate high specific conductance (219–1012 uS cm^−1^), Chloride (44–170 mg L^−1^), Nitrate-N (1.6–34 mg L^−1^), Ammonia-N (< 0.01–1.7 mg L^−1^), and Total Phosphorus (0.013–0.35 mg L^−1^) (NHDES 2020; NHDES 2022a; NHDES 2023; NHDES 2024; NHDES 2025). Monitoring of surface water outputs from the wetland has demonstrated consistently high total phosphorus, averaging 0.052 mg L^−1^ in 2022, and continuing at high levels through 2025 (0.035–0.047 mg L^−1^) (NHDES 2022c).

### Soil sampling

Within each of the five wetlands, three sampling sites were established, and three samples were taken from surface (0–10 cm) and one subsoil (below 47–103 cm, depending on the site) from the three different sampling sites. The three surface soil samples were combined into one composite sample after weighing, mesuring and drying. This resulted in three composite surface soil samples and three subsoil samples per wetland, with the exception of the Woods Crossing site which had only one subsoil sample. *In situ* measurements of pH and specific conductance were taken at the time of soil sampling (Table 1).

### Initial Characterization and Analytical Methods

Soil samples were dried at 60°C for four to five days until a constant weight was achieved. Bulk density was determined by dividing the dry mass by the initial volume (Table 2) on the subsamples at each of the three sample sites in each wetland. Dried samples were ground to pass through a 250 μm sieve.

Soil organic matter was determined using the loss-on-ignition (LOI) method, where soils were dried, ground and then combusted at 550°C for 3 hours. (Richardson and Reddy 2013).

Total phosphorus (TP) was measured on 0.2-0.5-gram oven-dried and ground samples which were ignited at 550°C for 3 hours. Following ignition, ash residues were dissolved in 6M hydrochloric acid followed by filtration through a 0.45 μm membrane filter. Total phosphorus concentrations were then determined via colorimetric analysis using the ascorbic acid-molybdate blue method (Richardson and Reddy 2013).

Composite soil samples were analyzed at the Penn State Agricultural Analysis Services Lab using the Mehlich-3 extraction method paired with inductively coupled plasma (ICP) spectroscopy for the determination of Mehlich-3 extractable aluminum (Al_M3_), Mehlich-3 extractable iron (Fe_M3_), and Mehlich-3 extractable phosphorus (P_M3_) (Wolf 2011).

### Phosphorus Characterization Methods

To directly evaluate soil phosphorus saturation and remaining retention capacity, two complementary phosphorus indices were calculated. The first was the phosphorus saturation ratio (PSR), the ratio between the amount of phosphorus present in the soil and the total capacity of the soil to retain phosphorus, calculated as:

$${\text{PSR}}_{\text{M}3}=\frac{\frac{{\text{P}}_{\text{M}3}}{31}}{\frac{{\text{Fe}}_{\text{M}3}}{56}+\frac{{\text{Al}}_{\text{M}3}}{27}}$$

where PSR is expressed as a molar ratio; P_M3_, Fe_M3_ and Al_M3_ are Mehlich-3 extractable concentrations (mg kg^−1^) of phosphorus, iron, and aluminum; and 31, 56, and 27 are the respective molar masses (g mol^−1^). This approach is similar to that described by Nair and Harris (Nair 2004); however, their analysis used a Mehlich-1 extraction. Only iron and aluminum are considered here because these minerals control inorganic P solubility in acid soils such as those found in the five wetlands under investigation (Reddy et al. 2005).

The second index used to characterize soil phosphorus status was soil phosphorus storage capacity (SPSC), which estimates the additional amount of phosphorus a soil can retain before reaching an environmental risk threshold. SPSC was calculated as:

$${\text{SPSC}}_{{\text{M3(mg kg}}^{-1})}=(\text{Threshold}\;{\text{PSR}}_{\text{M3}}-{\text{Soil}}_{\text{M3}})\;\left[\frac{{\text{Fe}}_{\text{M3}}}{56}+\frac{{\text{Al}}_{\text{M3}}}{27}\right]\times 31\times 1.5$$

where SPSC_M3_ is expressed in mg kg^−1^; threshold PSR_M3_ represents a critical change value above which phosphorus release to the environmental increases significantly (a value of 0.10 is commonly used for northeastern soils (Dari 2018); soil PSR_M3_ is the measured saturation ratio; Fe_M3_ and Al_M3_ are Mehlich-3 extractable concentrations of iron and aluminum (mg kg^−1^); 31, 56, and 27 are the molar masses of phosphorus, iron and aluminum respectively, and 1.5 is a correction factor accounting for the lower extractive strength of Mehlich-3 relative to ammonium oxalate for amorphous iron and aluminum (Chakraborty 2021).

### Data Analysis

Single factor ANOVA followed by Bonferroni post testing was utilized to establish a baseline for the “unaffected” control group of Bean Hill, Mid Bower Brook, Upper Bower Brook, and Woods Crossing. This was necessary to determine the natural background variation present in these sites.

To determine if the WWTF and RIB have driven soil parameters completely outside the statistical boundaries of the natural variation seen in the surface soils of the unaffected wetlands (Table 3), the data from the four unaffected wetlands was pooled prior to Welch’s t-testing versus the affected wetland, Beaver Dam.

## RESULTS

In the surface soils, there was a statistically significant difference in Al_M3_ and Fe _M3_ as well as PSR among these four unaffected wetlands (see Table 3). Subsequent Bonferroni post testing (p < 0.008) determined no statistically significant difference in Fe _M3_ and a statistically significant difference in the Al_M3_ (p = 0.004) and phosphorus saturation ratio (p = 0.002) between Bean Hill and Mid Bower Brook. There was no significant difference in soil pH, P_M3_, SPSC, TP (mg kg^−1^), TP (kg ha^−1^), or LOI.

A comparison of the subsoils (Table 4) collected from three of the wetlands unaffected by the WWTF and RIB (Woods Crossing was excluded due to insufficient sample size) by single factor ANOVA resulted in a statistically significant difference in the soil pH and LOI. Subsequent Bonferroni post testing (p < 0.017) determined no statistically significant difference in either soil pH or LOI. There was no significant difference in P_M3_, Al_M3_, Fe_M3_, PSR, SPSC or TP (mg kg^−1^).

The results from Tables 3 and 4 indicate that while there is no natural variation between the subsoils in the wetlands unaffected by the WWTF and the RIB there is some natural variation between the surface soils. The relatively low concentration of phosphorus sorbing Al_M3_ and Fe_M3_ in the Bean Hill wetland results in a PSR above the threshold value of 0.10 (PSR = 0.20) commonly used for northeastern soils (Dari 2018) and a negative SPSC indicating Bean Hill is the only unaffected wetland acting as a P-source. While Middle Bower Brook, Upper Bower Brook and Woods Crossing are acting as a P-sink and still actively absorbing phosphorus, Bean Hill is naturally saturated and is actively leaching phosphorus without input from an outside source.

While minor localized difference in surface Al_M3_ and PSR were present among the four unaffected wetlands, pooling of these sites captures the natural range of regional background variability and establishes a robust baseline for comparison to the wastewater-affected BDM. Welch’s t-testing of the wastewater-affected BDM surface soil compared to the pooled unaffected wetlands’ surface soil (Table 5) resulted in significantly higher P_M3_ and TP (mg kg^−1^) in the unaffected wetland and significantly higher LOI in the affected wetland. There was no statistically significant difference in the soil pH, Al_M3_,Fe_M3_, TP(kg ha^−1^), PSR or SPSC between the affected and unaffected wetland surface soil.

A comparison of the subsoils between the affected and unaffected wetlands (which includes the single sample from Woods Crossing) tells a different story (Table 6). The soil pH, P_M3_, Al_M3,_ PSR, and LOI were all significantly higher in the unaffected wetland. There was no statistically significant difference in the Fe_M3_, SPSC or TP (mg kg^−1^) between the subsoils in the affected and unaffected wetlands.

A further breakdown of the individual surface soil samples from BDM show that the soil sample taken from the part of the wetland that is no longer receiving wastewater effluent is behaving as a P-sink (PSR < 0.10, SPSC > 0) while the two soil sample sites acting as a P-source (PSR > 0.10, SPSC < 0) came from the part of the wetland that is still subject to wastewater inputs (Fig. 1). The area of the wetland with continued wastewater input has reached its saturation limit and the input of additional phosphorus is being washed though the wetland while the area near the historic discharge continues to maintain its retention capacity.

## DISCUSSION

We hypothesized that the wastewater-affected BDM would exhibit a higher phosphorus saturation ratio (PSR) and a lower soil phosphorus storage capacity (SPSC) than the unaffected wetlands. We also hypothesized that BDM would have a higher total phosphorus concentration in the surface soils. This was predicated on the assumption that over 40 years of treated high-phosphorus wastewater inputs would saturate available iron and aluminum sorption sites within BDM. However, our results rejected both hypotheses. A comparison of the surface soils between wastewater-affected BDM and unaffected wetlands shows no significant difference in the PSR or SPSC and found significantly *lower* P_M3_ and TP in the affected BDM (Table 5). In the subsoils (Table 6), the affected BDM also showed statistically *lower* P_M3_, as well as a *lower* PSR, while the SPSC and the TP showed no significant difference from the unaffected wetlands.

One of the most important mechanisms for phosphorus retention in wetlands is accretion of organic matter in the surface soils. The results in Table 5 contradict our hypothesis that soil TP would be higher in the wastewater-affected BDM as the total phosphorus in the organic surface soils was found to be lower than in the unaffected wetlands; this range of results is consistent with those found for enriched marshes by Kadlec (2019). However, BDM was also found to contain a significantly higher percent of organic matter resulting in a lower bulk density of its surface soil. Additionally, BDM has deeper layers of organic matter. Measuring phosphorus as a concentration by dry mass (mg P per kg of soil) does not consider the higher percentage of organic matter, lower bulk density and deeper layers of organic matter of the BDM, which may be causing a dilution effect. Correcting for bulk density and soil volume also suggest there is less TP in the surface soils of BDM when considered on a mass per area basis, although this difference is not significant, when compared to the unaffected wetlands.

The surface soils of BDM may also have lower TP (mg kg^−1^ and mg ha^−1^) even with the continued phosphorus input from the WWTF due to the invasion and rapid expansion of colonies of *Phragmites australis*. Based on examination of aerial photographs from 1954–2025 (Koning unpublished data), *P. australis* appears to have first colonized the wetland when wastewater discharge first began in 1965. These clonal colonies expanded but did not form new colonies, until the location of wastewater discharge (now through groundwater) changed, after 2009. New colonies appeared exactly where groundwater discharge surfaced in the wetland (Koning and Stroble 2022), covering approximately 14% of the wetland by 2025.

*P. australis* are known to invade and demonstrate vigorous growth and expansion in areas with elevated nutrients and moderate amounts of other pollutants (Kettenring et al. 2011; Ge 2017; Price et al. 2014). Because *P. australis* has very large above and below-ground biomass, it is possible that the additional extractable phosphorus is being stored in the biomass rather than the soils as it expands every year. Additionally, it has been found that *P. australis* biomass production, nutrient content of plant tissue, shoot/root ratios, and translocation rates were much greater in nutrient-rich wetlands than in the natural wetlands (Meuleman et al. 2002). A careful mass balance of phosphorus inputs, outputs, and storages to BDM as well as testing of the plant tissue for phosphorus would be necessary to fully explain the results found here.

The other set of mechanisms likely to be sequestering phosphorus in the soils is the formation of inorganic complexes with aluminum or iron. The aluminum-phosphorus complexes are sensitive to pH. BDM exhibited a significantly lower soil pH, and lower P_M3_ and Al_M3_. The solubility of aluminum in soils increases with increasing acidity, changing to the soluble Al^+ 3^ form between pH 5.5 and 4.5. (AgSource Laboratories 2024). In BDM where the soil pH is 4.07, the aluminum is highly soluble and can be washed out of the subsoil layer by the hydraulic flushing from the RIBs and other inputs, resulting in the statistically low Al_M3_ seen in Table 6, which is then unavailable to adsorb the phosphorus. In addition, chloride inputs from the WWTF may also interfere with the adsorption of phosphorus with aluminum or iron (Jin et al. 2013). Thus, the extractable phosphorus remains mobile and tends to be flushed out of the subsoil along with the aluminum, resulting in lower levels of extractable P_M3_.

The low soil pH in BDM subsoil may result from a combination of several factors including the chemical processes from the WWTF effluent. The subsoil pH may be lower because the effluent from the RIB is high in Nitrate-N and Ammonia-N (NHDES 2020; NHDES 2022a; NHDES 2023; NHDES 2024; NHDES 2025). Nitrification releases hydrogen ions causing acidification of the subsoil. Likewise, as the *P. australis* take up nutrients, they release hydrogen ions into the soil matrix to maintain electrical balance adding to the acidification of the subsoil (Hinsinger 2003).

While the concentration of both P_M3_ and Al_M3_ are lower in the affected BDM subsoil compared to the unaffected wetlands, the concentration of Fe_M3_ is greater in BDM, although the difference is not statistically significant, causing the calculated PSR to decrease compared to the unaffected wetlands. Comparison of the SPSC of the affected BDM to the unaffected wetland remains similar because while there are less overall phosphorus sorption sites in BDM there is also less extractable phosphorus to bind to those sites keeping the remaining storage capacity similar to the unaffected wetlands with more phosphorus sorption sites but also a greater concentration of extractable phosphorus.

These results suggest that any additional extractable phosphorus from the WWTF and RIB is not forming into aluminum-phosphate and iron-phosphate complexes (which would raise the PSR and lower the SPSC) but is instead being sequestered in the *P. australis* away from the surface soil before it can be saturated into the underlying iron and aluminum complexes, thus keeping the PSR lower and the SPSC higher and in line with the values from the unaffected wetlands.

We also found that there was some spatial variation within the soils of BDM, indicating that the samples taken from the area still receiving wastewater input from the RIB have reached their saturation limit, whereas the soil near the historic (not ongoing) discharge continues to maintain its retention capacity. This may explain the continued high total phosphorus being released in the surface water from this wetland, discharging to the lake. Additional sampling and analysis would be necessary to verify these findings.

## CONCLUSION

This study evaluated the effect of 40 years of wastewater effluent input on a 7.3 ha wetland in southwestern New Hampshire, USA. Our findings rejected our original hypotheses of higher TP, higher PSR, and lower SPSC in the wastewater-affected BDM wetland when compared to the natural baseline from the pooled data of four unaffected wetlands nearby. In BDM surface soils, the rapid expansion of *Phragmites australis* coinciding with the change in wastewater flow path through BDM suggests the added extractable phosphorus is being sequestered into the expanding biomass or being diluted by rapid accretion of deep layers of organic matter rather than accumulating in inorganic aluminum and iron complexation. Conversely, in BDM subsoils, soil acidification and chloride input from the wastewater effluent are likely increasing the solubility of aluminum and destabilize the sorption sites allowing the phosphorus to remain highly mobile in the soil matrix.

Over four decades of effluent loading, the wastewater-affected BDM has taken in and stored a large amount of phosphorus in its expansive organic matter layer. However, its ability to adsorb phosphorus through inorganic iron and aluminum sorption is limited particularly around the area of this wetland where wastewater input is on-going resulting in a transition of a portion of this wetland from a phosphorus sink to a source and allowing phosphorus to discharge downstream to a nearby lake.

To better understand these underlying processes, direct testing of the *P. australis*, both the above ground biomass and the belowground roots and rhizomes and mass-balance calculations, are necessary to quantify the role of *P. australis* in phosphorus sequestration. Additionally, further high-resolution spatial sampling across the historic surface discharge and current discharge regions of BDM is necessary to map localized saturation boundaries within this wastewater affected wetland.

## Figures and Tables

**Figure 1:**
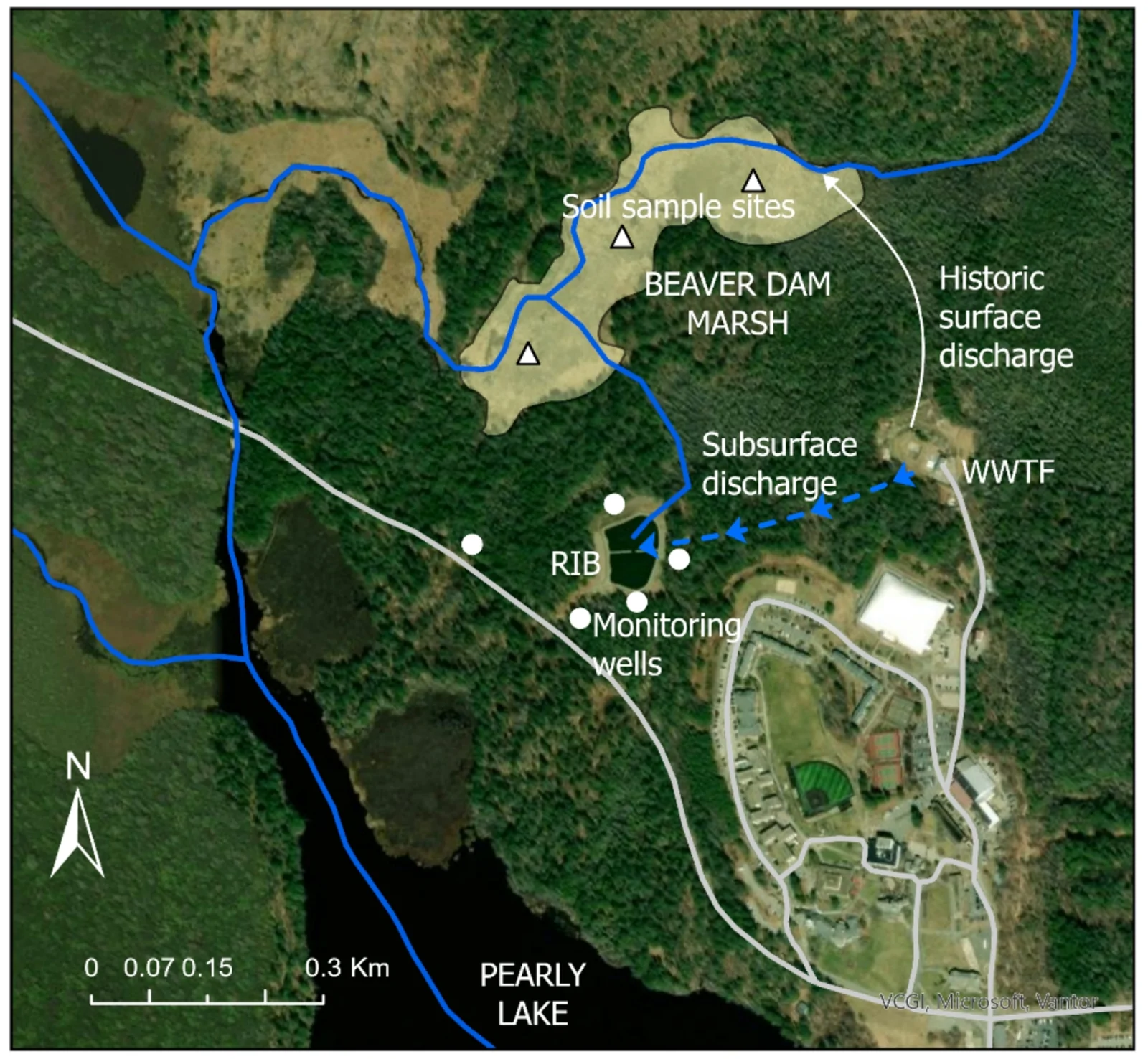
Map of wastewater-affected wetland, Beaver Dam Marsh. RIB=Rapid Infiltration Beds; WWTF=Wastewater Treatment Facility

**Figure 2:**
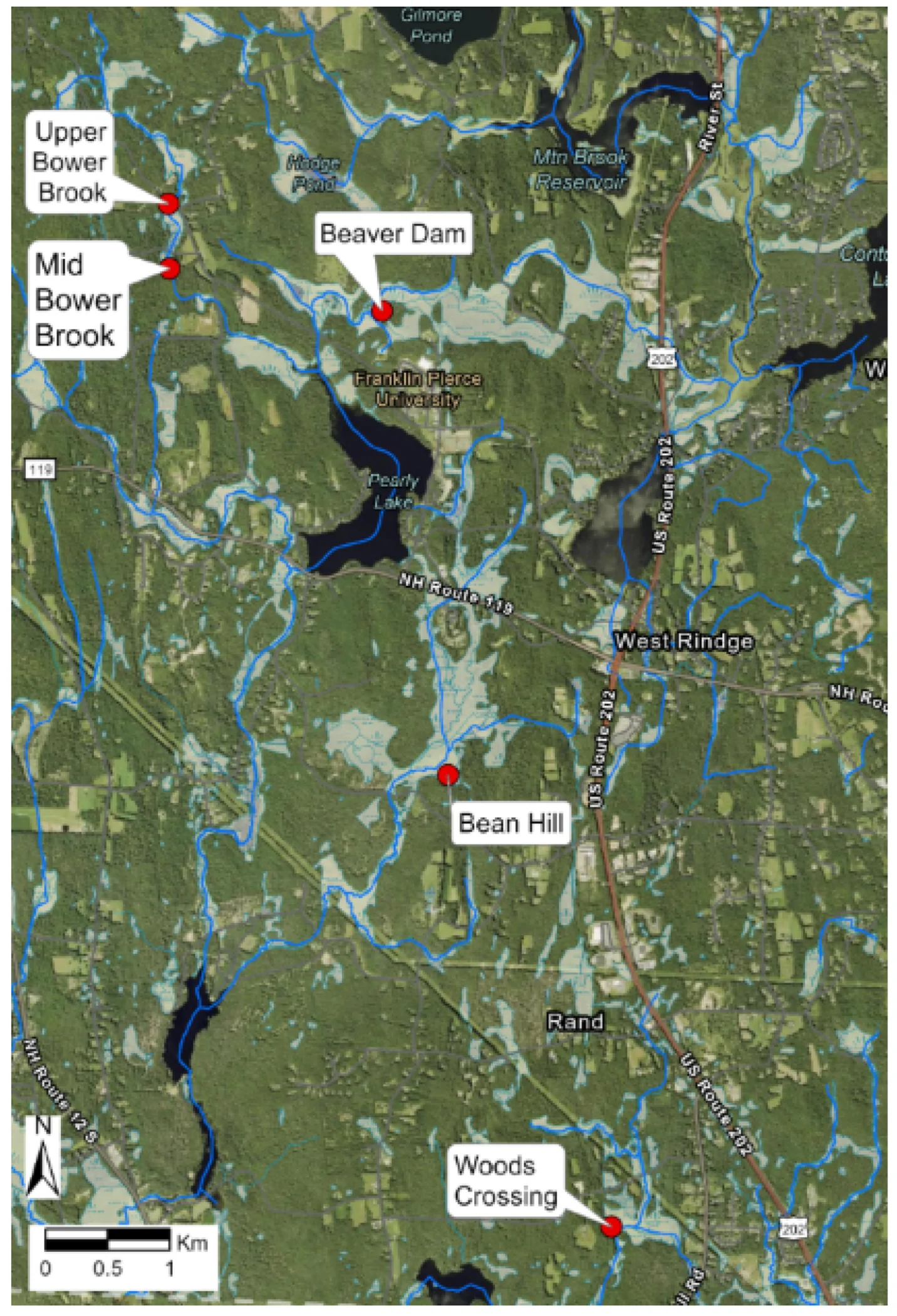
Study site locations, Rindge NH USA

**Table 1:** Wetland Classification, Vegetation and Water Quality.

Wetland, coordinates	Size (ha)	NWI classification	Dominant vegetation	Surface water pH	Surface water Specific Conductance (μS _cm_−1_)_
Beaver Dam 42.788, −72.062 (wastewater impacted)	7.3	PEM1E	*Calamagrostis canadensis* *Typha latifolia* *Carex stricta* *Frangula alnus* *Lysimachia terrestris* *Phragmites australis* *Sphagnum spp*.	5.14	240
Bean Hill 42.752, −72.060	11.3	PSS1E/PEM1E	*Calamagrostis canadensis Chamaedaphne calyculata Sphagnum spp*.	3.83	90
Mid Bower Brook 42.792, −72.082	0.70	PSS1E	*Calamagrostis canadensis* *Calla palustris,* *Carex stricta, C. lacustris* *Lysimachia terrestris* *Sagittaria latifolia* *Scirpus cyperinus* *Spiraea tomentosa*	4.87	48
Upper Bower Brook 42.795, −72.083	3.5	PEM1E/PEM2F	*Calamagrostis canadensis* *Calla palustris, Carex stricta, Chamaedaphne calyculata, Frangula alnus* *Lysimachia terrestris* *Scirpus cyperinus* *Spiraea tomentosa* *Sphagnum spp*	4.28	24
Woods Crossing 42.723, −72.039	4.0	PEM1E	*Carex stricta, Scirpus cyperinus,* *Calamagrostis canadensis, Chamaedaphne calyculata, Spiraea tomentosa, Spiraea alba*	4.61	220

**Table 2:** Soil Characteristics

Wetland	Soil type	Average Depth of organic layer (cm)	Subsoil texture	Average Bulk density 0–10 cm (g cm^−3^)	Average LOI 0–10 cm (%)	Average Soil pH 0–10 cm
Beaver Dam	Borohemist ponded	115	Clay, silt, fine sand	0.057	87.52	4.56
Bean Hill	Borohemist ponded	88	Sand, coarse sand	0.052	89.79	4.62
Mid Bower Brook	Borohemist ponded	69	Clay, silt, sand, coarse sand, gravel	0.093	76.52	4.50
Upper Bower Brook	Borohemist ponded	85	Sand, coarse sand, gravel	0.085	79.25	4.50
Woods Crossing	Borohemist ponded	93	Sand, coarse sand, gravel	0.058	73.57	4.19

**Table 3:** Unaffected wetland surface soil analysis results, average (N = 3). Values with the same superscript in each column were not significantly different based on Bonferroni post-testing (p < 0.008)

Bean Hill	Sample Depth (cm)	Soil pH	P_M3_ (mg kg^−1^)	Al _M3_ (mg kg^−1^)	Fe _M3_ (mg kg^−1^)	PSR	SPSC (mg kg^−1^)	TP (mg kg^−1^)	TP (kg ha^−1^)	LOI (%)
	
	**0–10**	**4.63^a^**	**45 ^a^**	**136 ^a^**	**127 ^a^**	**0.20 ^a^**	**−34 ^a^**	**1171 ^a^**	**521 ^a^**	**89.8 ^a^**

Mid Bower Brook	0–10	4.50 ^a^	61 ^a^	659 ^b^	352 ^a^	0.07 ^b^	51 ^a^	1476 ^a^	996 ^a^	76.5 ^a^

Upper Bower Brook	0–10	4.50 ^a^	39 ^a^	298 ^ab^	148 ^a^	0.09 ^ab^	6 ^a^	1287 ^a^	925 ^a^	79.3 ^a^

Woods Crossing	0–10	4.20 ^a^	64 ^a^	525 ^ab^	236 ^a^	0.11 ^ab^	15 ^a^	1350 ^a^	732 ^a^	73.6 ^a^

*ANOVA p-value*		*0.27*	*0.072*	*0.039*	*0.0075*	*0.022*	*0.089*	*0.41*	*0.47*	*0.36*

**Table 4:** Unaffected wetlands subsoil analysis results, average (N = 3).* Values with the same superscript in each column were not significantly different based on Bonferroni post-testing (p < 0.017)

Bean Hill	Sample Depth (cm)	Soil pH	P_M3_ (mg kg^−1^)	Al _M3_ (mg kg^−1^)	Fe _M3_ (mg kg^−1^)	PSR	SPSC	TP (mg kg^−1^)	LOI (%)
	
	88–98	4.10 ^a^	24 ^a^	524 ^a^	225 ^a^	0.03 ^a^	73 ^a^	460 ^a^	3.3 ^a^

Mid Bower Brook	47–57	5.00 ^a^	96 ^a^	982 ^a^	48 ^a^	0.08 ^a^	29 ^a^	705 ^a^	7.3 ^a^

Upper Bower Brook	65–75	4.77 ^a^	108 ^a^	940 ^a^	97 ^a^	0.08 ^a^	8 ^a^	997 ^a^	20.2 ^a^

Woods Crossing	93–103	3.60	8	359	350	0.01	79	379	5.1

*ANOVA p-value*		*0.049*	*0.40*	*0.12*	*0.18*	*0.41*	*0.70*	*0.37*	*0.027*

* Woods Crossing was excluded from the analysis due to insufficient sample size (n = 1)

**Table 5:** Surface soil analysis results, average.

Affected Wetland, BDM n = 3	Sample Depth (cm)	Soil pH	P_M3_ (mg kg^−1^)	Al _M3_ (mg kg^−1^)	Fe _M3_ (mg kg^−1^)	PSR	SPSC (mg kg^−1^)	TP (mg kg^−1^)	TP (kg ha^−1^)	LOI (%)
	
	0–10	4.57	40	275	185	0.11	3	995	650	87.5

Unaffected Wetlands n = 12	0–10	4.46	52	405	216	0.12	9	1321	793	79.8

*Welch’s T-Test p-value*		*0.56*	*0.038*	*0.31*	*0.38*	*0.90*	*0.79*	*2.3x10^−4^*	0.24	*0.038*

**Table 6:** Subsoil analysis results, average.

Affected Wetland, BDM n = 3	Depth of OM in sample location (cm)	Soil pH	P_M3_ (mg kg^−1^)	Al _M3_ (mg kg^−1^)	Fe _M3_ (mg kg^−1^)	PSR	SPSC (mg kg^−1^)	TP (mg kg^−1^)	LOI (%)
	
	111–120	4.07	12	267	463	0.02	66	553	1.6

Unaffected Wetlands n = 10	69–93	4.51	69	770	146	0.06	41	686	9.8

*T-Test p-value (subsoil)*		*0.048*	*0.042*	*0.0084*	*0.058*	*0.029*	*0.42*	*0.35*	*0.016*

## Data Availability

The datasets generated during and/or analyzed during the current study are available from the corresponding author on reasonable request.
